# The complete mitochondrial genome of the *Perinereis linea* (Polychaeta: Nereididae) and Nereididae phylogenetic implications

**DOI:** 10.1080/23802359.2026.2654107

**Published:** 2026-04-09

**Authors:** Xue-Feng Song, Yan-Qing Wu, Jia-Yuan Xu, Zhi-Xing Su, Shuai Zhou, Bian-Bian Zhang, Li-Guo Yang, Xiao-Kang Lv

**Affiliations:** East China Sea Fisheries Research Institute, Chinese Academy of Fishery Sciences, Shanghai, China

**Keywords:** *Perinereis linea*, mitochondrial genome, nereididae, phylogenetic analyses

## Abstract

*Perinereis linea* is a widespread marine polychaete, has its complete mitochondrial genome reported here from Dongying, China. The 15,854 bp genome comprises 13 PCGs, 2 rRNAs, 22 tRNAs, and a control region, with typical annelid gene order and 64.63% AT bias. Phylogenetic analyses of 20 annelids places *P. linea* close to *Perinereis aibuhitensis*, clarifying nereidid evolution. This genome aids species identification and evolutionary studies.

## 1. Introduction

*Perinereis linea* (Treadwell, 1936) is an important marine polychaete species that predominantly in-habits intertidal zones with muddy and sandy sediments (Treadwell 1936). Despite its ecological significance, the genetic information of *P. linea* remains limited (Yang et al. 2024). Understanding the mitochondrial genome of *P. linea* can provide insights into its evolutionary relationships and help in the conservation of marine biodiversity (Marnis et al. 2024). The mitochondrial genome is a valuable tool for species identification and phylogenetic analysis due to its conserved structure and maternal inheritance pattern (Liu et al. 2025). In this study, we aim to determine the complete mt genome of *P. linea* and analyze its phylogenetic relationships within the family Nereididae.

Marine ecosystems are complex and diverse, with a wide variety of species interacting in intricate ways (Qian et al. 2022). Polychaetes, in particular, are an important group of marine worms that contribute significantly to the biodiversity and functioning of these ecosystems (Vinithkumar et al. 2008). *P. linea*, a member of the family Nereididae, is known for its ability to thrive in various marine environments, from shallow coastal waters to deeper offshore areas (Tosuji et al. 2019). Its presence is often indicative of a healthy and diverse ecosystem, making it an important bioindicator species (Villalobos-Guerrero 2019).

The ecological role of *P. linea* is multifaceted. It serves as a food source for many marine animals, including fish, birds, and other invertebrates. Additionally, it plays a crucial role in nutrient cycling and sediment stabilization (Fallon and Austin 1967). Its burrowing activities help to aerate the sediment, facilitating the exchange of nutrients and oxygen between the water column and the seafloor (Fischer 1975). This, in turn, supports the growth of other marine organisms and contributes to the overall health of the ecosystem.

Despite its ecological importance, the genetic information of *P. linea* is still limited. Previous studies have focused mainly on its morphology, ecology, and distribution, with little attention given to its molecular characteristics (Joo et al. 2020; Dong et al. 2019). The mitochondrial genome, however, offers a wealth of information that can help fill this gap. It is highly conserved in structure and sequence, making it an ideal tool for phylogenetic analysis and species identification (Kim et al. 2015). Moreover, its maternal inheritance pattern ensures that it is passed down from mother to offspring without recombination, providing a clear and uninterrupted record of evolutionary history.

## 2. Materials and methods

**Figure 1.**
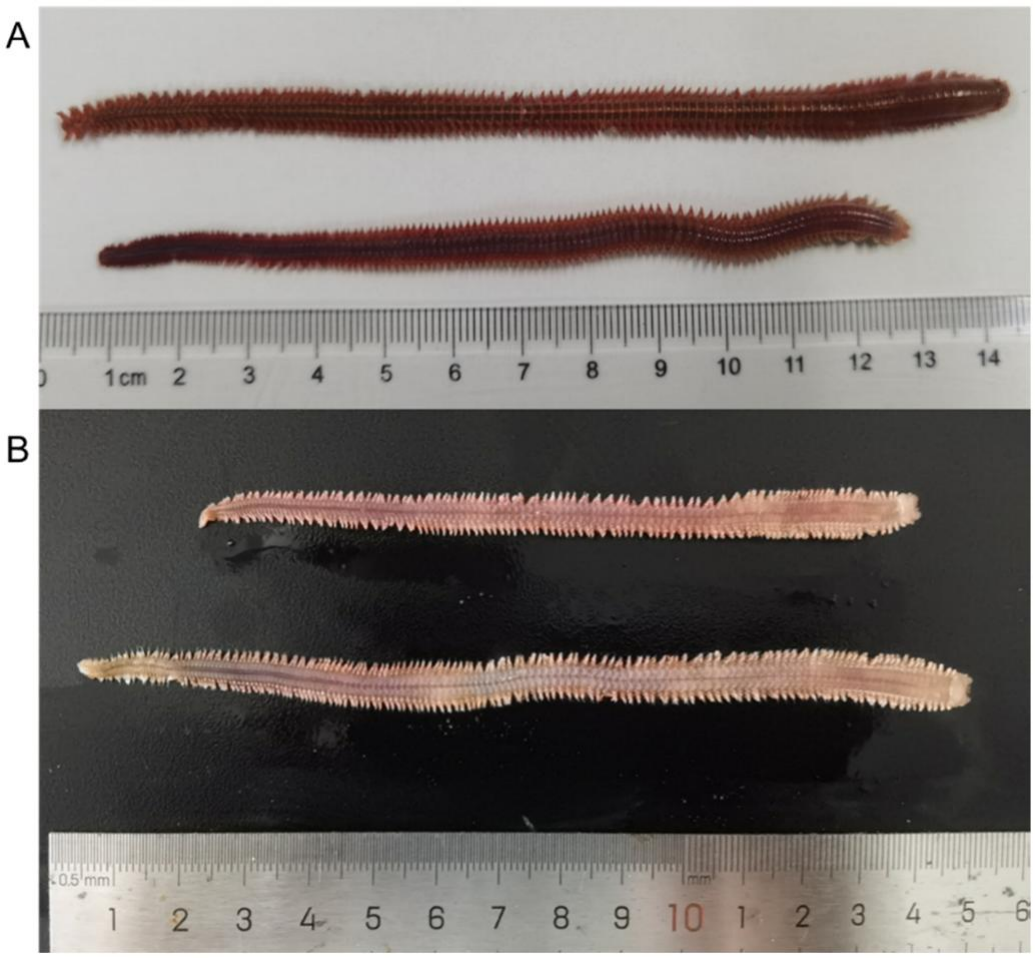
Reference image of *Perinereis linea*. (A) Dorsal view. (B) Ventral view. Both photographs were taken by the author of this article, Li-Guo Yang.

Samples of *P. linea* were collected from Dongying City, Shandong Province (118°55′13″ E, 37°30′1″ N) on April 6, 2024. The *P. linea* were preserved in DNA protective solution (Qiagen) and stored at −80 °C in the Ninghai Fishery Innovation Research Center, Ningbo, Zhejiang Province, until further analysis. The collection site was chosen based on their ecological significance and the known distribution of *P. linea*. A total of 50 specimens were collected, and the three selected individuals were combined into a single pooled sample for DNA extraction and sequencing. The specimens and genomic DNA are kept at the East China Sea Fisheries Research Institute, Chinese Academy of Fishery Sciences, Ninghai, China (voucher code: Ninghai20240406; contact person: Li-Guo Yang, yangliguo16888@163.com) (Figure 1).

Mitochondrial genome maps were constructed using OGDRAW (https://chlorobox.mpimp-golm.mpg.de/OGDraw.html, accessed on 7 Dec 2024). Tandem repeats were identified using the vmatch v2.3.0 software (https://www.vmatch.de, accessed on 7 Dec 2024) in combination with Perl scripts, with parameters set as follows: minimum length = 20 bp, hamming distance = 3. Homologous gene pairs were aligned using the mafft v7.427 software (https://mafft.cbrc.jp/alignment/software/, accessed on 8 Dec 2024), and the Ka and Ks values for each gene pair were calculated using the KaKs_Calculator v2.0 software (https://sourceforge.net/projects/kakscalculator2/, accessed on 11 Dec 2024) with the Maximum Likelihood with Whelan and Goldman model (MLWL) method. Homologous gene sequences from different species were globally aligned using the mafft software in–auto mode, and the pi value for each gene was calculated using DnaSP v6.12.03 (http://www.ub.edu/dnasp, accessed on 1 Feb 2025). The CGVIEW software (https://github.com/paulstothard/cgview, default parameters, accessed on 2 Feb 2025) was used for comparative analysis of mitochondrial genome structures among closely related species. Genome alignment was performed using the Mauve software (https://darlinglab.org/mauve/mauve.html, accessed on 3 Feb 2025) with default parameters.

Whole genome phylogenetic analysis was performed by setting the circular sequences to the same starting point. The MAFFT v7.427 software (–auto mode) was used for multiple sequence alignment of interspecies sequences, and the trimAl (v1.4.rev15) software was used to remove unreliable regions of the sequence alignment. Under the Bayesian information criterion, the jModelTest v2.1.10 software was used to identify the optimal nucleotide substitution model. The RAxML v8.2.10 software (https://cme.h-its.org/exelixis/software.html, accessed on 5 Feb 2025) was used to con-struct a maximum - likelihood phylogenetic tree with the GTRGAMMA model and rapid Bootstrap analysis (bootstrap = 1000).

## 3. Results

**Figure 2.**
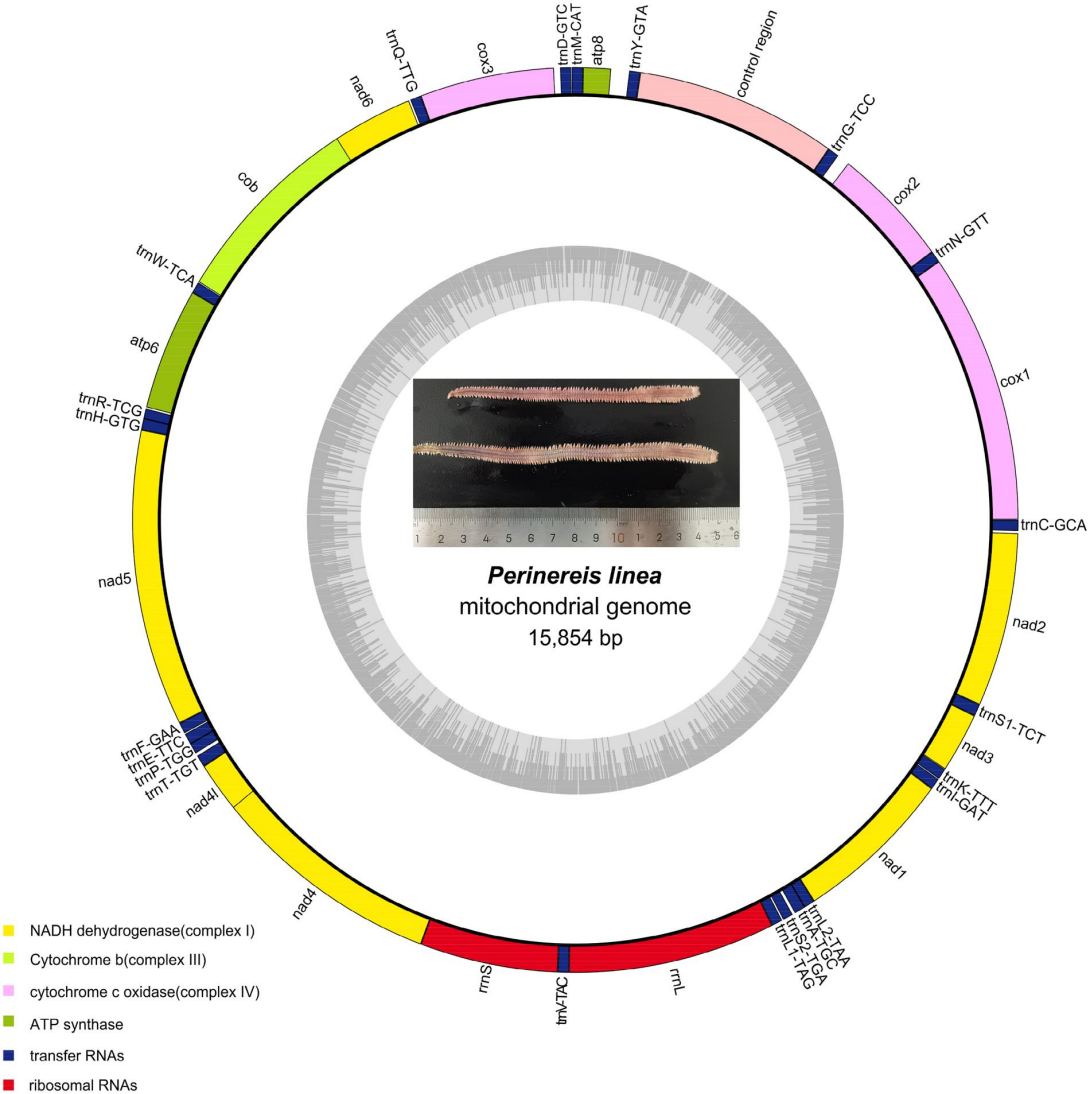
Mitochondrial genome structure of *P. linea.*

The complete mt genome of *P. linea* is 15,854 bp in length and contains 37 genes, including 13 protein-coding genes (PCGs), 2 rRNAs, 22 tRNAs, and a control region (GenBank accession number PV573995). The gene arrangement is consistent with the typical annelid MT genome structure. The base composition of the mt genome shows a AT bias, with an average AT content of 64.63% (A: 29.44%, G: 14.12%, C: 21.25%, T: 35.18%). The AT bias is a common feature of mitochondrial genomes in many species, reflecting the high proportion of adenine and thymine nucleotides compared to guanine and cytosine (Figures 2 and S1).

The initiation and termination codons of the PCGs were also analyzed. Most PCGs start with the typical ATG initiation codon, incomplete stop codons were detected in some genes, such as cox3 and nad5, which is a common feature in many mitochondrial genomes. It is assumed that the termination codon is completed by polyadenylation during post-transcriptional processing.

**Figure 3.**
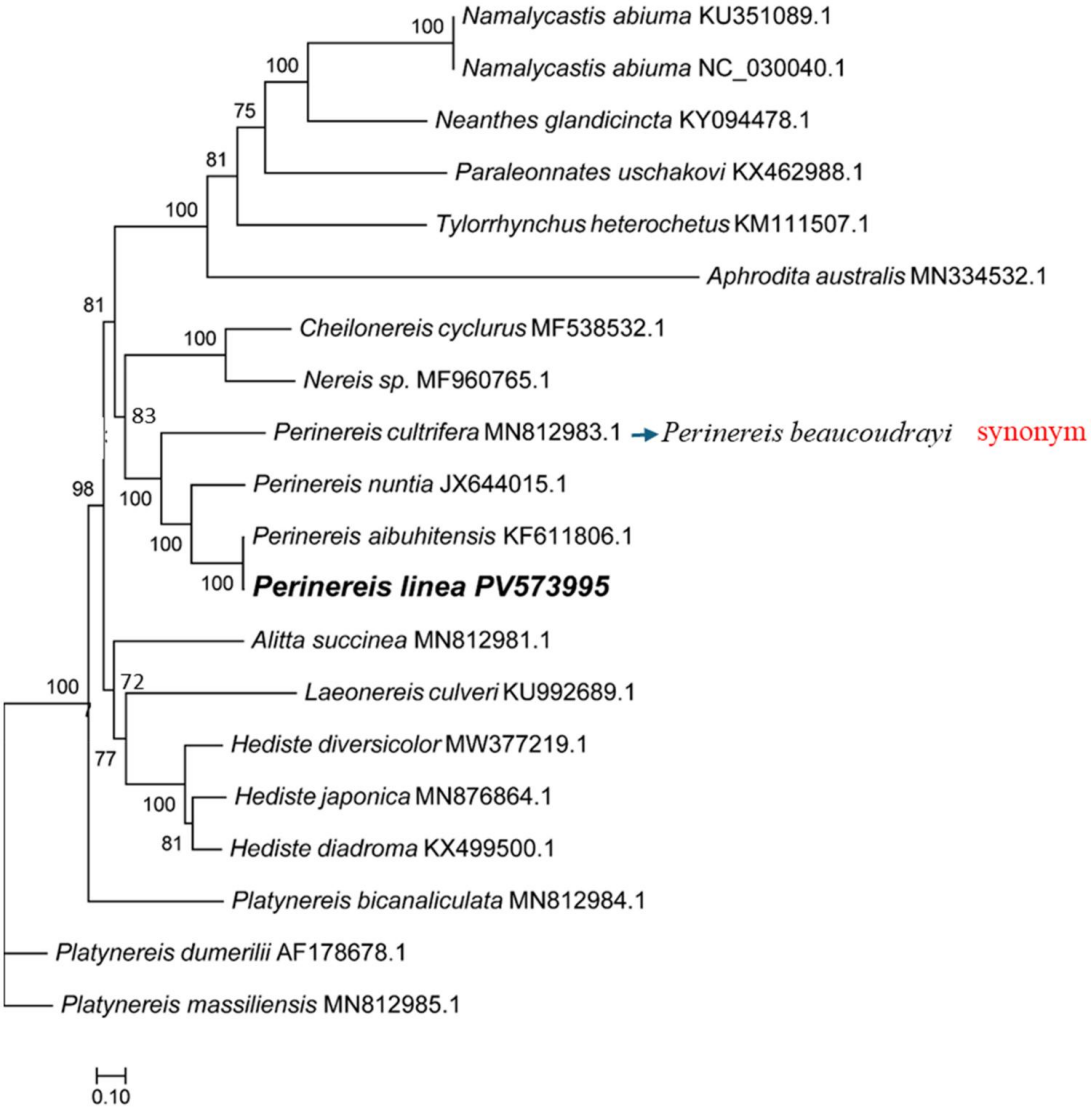
Maximum-likelihood phylogenetic tree based on complete mitochondrial genome sequences of Nereididae species. The following sequences were used: *Namalycastis abiuma* KU351089.1 (Lin et al. 2016), *Namalycastis abiuma* NC_030040.1 (Lin et al. 2016), *Neanthes glandicincta* KY094478.1 (Lin et al. 2017), *Paraleonnates uschakovi* KX462988.1 (Park et al. 2016), *Tylorrhynchus heterochetus* KM111507.1 (Direct submission), *Aphrodita australis* MN334532.1 (Wang et al. 2019), *Cheilonereis cyclurus* MF538532.1 (Park et al. 2017), *Nereis sp*. MF960765.1 (Kim et al. 2017), *Perinereis cultrifera* MN812983.1, synonym: *Perinereis beaucoudrayi* (Villalobos-Guerrero et al. 2024), *Perinereis nuntia* JX644015.1 (Tosuji et al. 2019), *Perinereis aibuhitensis* KF611806.1 (Kim et al. 2015), *Perinereis linea* PV573995 (This study), *Alitta succinea* MN812981.1 (Villalobos-Guerrero et al. 2024), *Laeonereis culveri* KU992689.1 (Direct submission), *Hediste diversicolor* MW377219.1 (Gomes-Dos-Santos et al. 2021), *Hediste japonica* MN876864.1 (Park et al. 2020), *Hediste diadroma* KX499500.1 (Kim et al. 2016), *Platynereis bicanaliculata* MN812984.1 (Gomes-Dos-Santos et al. 2021), *Platynereis dumerilii* AF178678.1 (Fischer 1975), *Platynereis massiliensis* MN812985.1 (Gomes-Dos-Santos et al. 2021). The tree was constructed using RAxML v8.2.10 with the GTRGAMMA substitution model and 1000 rapid bootstrap replicates.

Phylogenetic analyses indicated that *P. linea* is closely related to *Perinereis aibuhitensis* (KF611806.1). The mt genome of *P. linea* grouped with those of other nereidid species, forming a monophyletic clade. The phylogenetic tree showed strong sup-port for the relationships among the species, with high bootstrap values and posterior probabilities. The results of the phylogenetic analysis are consistent with previous studies on the evolutionary relationships within the family Nereididae (Figure 3).

## 4. Discussion and conclusions

The mitochondrial genome of *Perinereis linea* is 15,854 bp in length and contains 37 genes, including 13 protein-coding genes (PCGs), two rRNA genes, 22 tRNA genes, and a control region. Its organization follows the typical annelid mitochondrial architecture, with a pronounced AT bias (64.63% AT content), consistent with patterns observed in other annelids (Zou et al. 2017). The control region, spanning 1,166 bp, contains conserved sequence blocks and tandem repeats, playing a critical role in replication and transcription regulation (Jiang et al. 2018).

The combined length of the PCGs is 11,017 bp, with an AT content of 63.5%, similar to that of related species (Hassanin et al. 2005). Codon usage analysis indicated Leu as the most frequent amino acid, a common feature among annelid mitochondrial genomes (Chen et al. 2002). Some PCGs, such as cox3 and nad5, exhibited incomplete stop codons, likely completed *via* post-transcriptional polyadenylation (D’Souza et al. 2018). The 22 tRNAs ranged from 57 to 69 bp, with high AT content (64.63%) that may influence secondary structure and translational efficiency (Heinrichs et al. 2025). Nucleotide polymorphism analysis revealed several SNPs, particularly in nad5, offering insights into genetic diversity and selective pressures (Han et al. 2025).

Our phylogenetic analysis, based on complete mitochondrial genomes, robustly confirms the placement of *Perinereis linea* within the genus *Perinereis* and provides strong support for the monophyly of the genus itself, reinforcing generic boundaries proposed in earlier molecular studies (Alves and Santos 2016). Beyond species-level placement, the tree topology reveals an intriguing pattern related to morphological character evolution: species bearing ‘broken’ bar paragnaths (*P. nuntia*, *P. linea*, and *P. aibuhitensis*) form a distinct, late-diverging subgroup, while *P. beaucoudrayi* with ‘entire’ bar paragnaths occupies a more basal position. This phylogenetic separation based on paragnath morphology is consistent with the findings of Alves and Santos (2016).

However, this pattern presents an interesting discordance with the comprehensive revision by Teixeira et al. (2024), who suggested that paragnath morphology may exhibit greater plasticity and homoplasy than previously recognized. The discrepancy between our mitogenome-based topology and their findings highlights the complexity of nereidid systematics and underscores the need for integrative approaches combining mitochondrial genomes with multilocus nuclear data and broader taxonomic sampling to fully resolve the evolutionary history of this challenging group.

## Supplementary Material

Supplemental Material

## Data Availability

The data supporting the findings of this study are available within the article. All relevant data, including datasets analyzed or generated during the study, are included in the submitted manuscript. No additional external datasets were used, and no data are withheld due to privacy or ethical restrictions.
